# Mortality rate and biomarker expression within COVID-19 patients who develop acute ischemic stroke: a systematic review and meta-analysis

**DOI:** 10.2144/fsoa-2021-0036

**Published:** 2021-05-11

**Authors:** Ahmed Yassin, Ansam Ghzawi, Abdel-Hameed Al-Mistarehi, Khalid El-Salem, Amira Y Benmelouka, Ahmed M Sherif, Nesrine BenhadjDahman, Nameer AlAdamat, Amine Jemel, Ahmed Negida, Mohamed Abdelmonem

**Affiliations:** 1 Division of Neurology, Department of Neurosciences, Faculty of Medicine, Jordan University of Science & Technology, Irbid, Jordan; 2 Faculty of Medicine, Yarmouk University, Irbid, Jordan; 3 Department of Public Health & Family Medicine, Jordan University of Science & Technology, Irbid, Jordan; 4 University of Algiers, Algeria; 5 Faculty of Human Medicine, Zagazig University, Sharkia, Egypt; 6 Faculty of Medicine of Tunis, University of Tunis El Manar, Tunisia; 7 Department of Internal Medicine, Faculty of Medicine, Jordan University of Science & Technology, Irbid, Jordan; 8 Department of Thoracic & Cardiovascular Surgery, Abderrahmen Mami Hospital, Tunisia; 9 School of Pharmacy and Biomedical Sciences, University of Portsmouth, Portsmouth, Hampshire, England; 10 Faculty of Medicine, Fayoum University, Egypt

**Keywords:** biomarkers, coronavirus, COVID-19, ischemic stroke, mortality rate, stroke

## Abstract

**Objective:**

To describe the mortality difference between acute ischemic stroke (AIS) and non-AIS groups within COVID-19 patients.

**Materials & methods:**

We included observational studies through September 2020 that categorized COVID-19 patients into two groups (with and without AIS).

**Results:**

Eight studies with a total sample size of 19,399 COVID-19 patients were included. The pooled risk difference showed that patients with COVID-19 who developed AIS had significantly higher mortality than those without AIS by a risk difference of 24% (95% CI: 0.10–0.39; p = 0.001). In two studies, the COVID-19+AIS group had significantly higher lymphocytes, procalcitonin and creatinine levels.

**Conclusion:**

Developing AIS significantly adds to the mortality of COVID-19. Timely interventions to manage those patients are strongly recommended.

## Lay abstract

We systematically searched for COVID-19 studies that categorized patients into two groups: with and without acute ischemic stroke (AIS). Of 5100 unique records, eight studies with a total of 19,399 COVID-19 patients were included. The overall mortality rate of COVID-19 patients who developed AIS was 29.6% compared with 2.6% in those without AIS. We therefore conclude that development of AIS increases the mortality rate of COVID-19, and recommend timely intervention for such patients.

The emergence of SARS-CoV-2 in China at the end of 2019 and its subsequent global spread caused unprecedented health and economic challenges. It is characterized by its rapid contagion, reaching more than 134 million confirmed cases, including more than 2.91 million deaths globally by 10 April  2021 [1]. The clinical presentation of COVID-19 varies, ranging from asymptomatic infection to severe complications, including acute respiratory distress syndrome, neurological and cardiovascular complications and even death [2–4].

COVID-19 is also associated with inflammatory coagulopathy that causes disseminated vascular obstructions, including but not limited to acute ischemic stroke (AIS) [5–7]. Several hypotheses have been proposed to explain the increased risk of AIS, such as alteration in the stability of atherosclerotic plaques [8], tissue hypoxia caused by inflammation [9], and the more recent concept of cerebral vasculitis [10]. Antiphospholipid antibodies have also been positive in severely infected patients with AIS, regardless of their genetic predisposition [5,6].

Although healthcare resources including personnel, hospital and intensive care unit beds and physicians should be directed toward caring for COVID-19 patients, this should not compromise the care of patients presenting with AIS [11]. A German nationwide cohort study using all hospitalized patients' administrative database showed that the absolute number of stroke admissions declined during the pandemic. However, patients presenting with AIS continued to receive tissue plasminogen activator and mechanical thrombectomy according to standards [12].

Patients with AIS are usually at higher risk of mortality and morbidity than healthy controls [13–16]. However, the mortality rates of COVID-19 patients who develop AIS compared with those without AIS remain unanswered. This systematic review and meta-analysis aimed to compare the mortality rates of COVID-19 patients who developed AIS with those who did not.

## Materials & methods

The study was conducted following the recommendations of the Preferred Reporting Items for Systematic Reviews and Meta-Analyses (PRISMA) checklist for systematic reviews [17]. The review underwent critical appraisal following the A MeaSurement Tool to Assess systematic Reviews 2 (AMSTAR 2) checklist [18].

### Search Strategy

We systematically searched PubMed, Scopus and Web of Science from database inception to August 2020. The search strategy was: (“brain” OR “cerebral” OR “cerebrovascular” OR “CNS” OR “large vessel” OR “central nervous system”) AND (“infarction” OR “occlusion” OR “thrombosis” OR “thrombotic” OR “cerebrovascular accident” OR “stroke”) AND (“coronavirus” OR “COVID” OR “SARS-COV 2” OR “severe respiratory distress syndrome”). A manual search was performed to minimize results bias by searching for relevant missed publications in included articles' references. The reference lists of the studies included in our study were searched to trace potential further studies. The search was updated in September 2020 to include articles published after the first search.

### Inclusion & exclusion criteria

Studies satisfying the following criteria were included:

- Study design: studies that were described as observational studies (cohort or case–control studies);
- Population: studies on COVID-19 patients categorized into two groups, with and without AIS, with confirmed temporality between COVID-19 and stroke;
- Outcome: studies reporting the mortality rate and demographic, clinical, and laboratory findings; and
- Language: only studies written in the English language were included.

We excluded *in vitro* or animal studies; reviews, thesis, books, conference papers or articles without available full texts; duplicated, overlapping, unreliably extracted or incomplete data; and preprints that were not peer-reviewed.

### Screening & study selection process

Two independent reviewers screened all records for eligibility. Eligibility screening was performed in two steps: in the first step, titles and abstracts were screened, and in the second step, full-text articles of the selected abstracts were retrieved and assessed for eligibility. Disagreements were resolved by discussion with a third reviewer.

### Data extraction

We extracted data on patients' demographic characteristics, past medical history, clinical presentation, laboratory values, treatments and clinical outcomes. Two independent reviewers extracted data to a uniform Microsoft Excel sheet. A third independent reviewer performed a further check on the retrieved data to confirm data accuracy. All disagreements were resolved through discussion.

### Assessing the risk of bias in the included studies

We used the Newcastle Ottawa Scale (NOS) to assess the risk of bias in the included observational studies. The NOS is a star-based score in which studies are judged on three domains: the selection of the study groups, the comparability of the groups and the ascertainment of either the exposure or outcome of interest for case–control or cohort studies, respectively.

### Data analysis

The differences between COVID-19 with AIS and COVID-19 without AIS groups were pooled as risk difference (RD) with the corresponding 95% CIs in the random effects meta-analysis model. For continuous outcomes, the standardized mean difference (SMD) between the study groups was pooled with the corresponding 95% CI in the random effect meta-analysis model. All analysis was done by the RevMan software for Windows (Review Manager Version 5.3. Copenhagen: The Nordic Cochrane Centre, 2014). A p-value <0.05 was considered for statistical significance.

### Heterogeneity

Heterogeneity was assessed by visual inspection of the forest plots to check the pooled estimates' overlapping 95% CIs. Heterogeneity was tested by the chi-square test and quantified by the I^2^ test. The outcomes were considered heterogeneous when the p-value was <0.1 and I^2^ >50%.

## Results

### Literature search results

**Figure 1.**
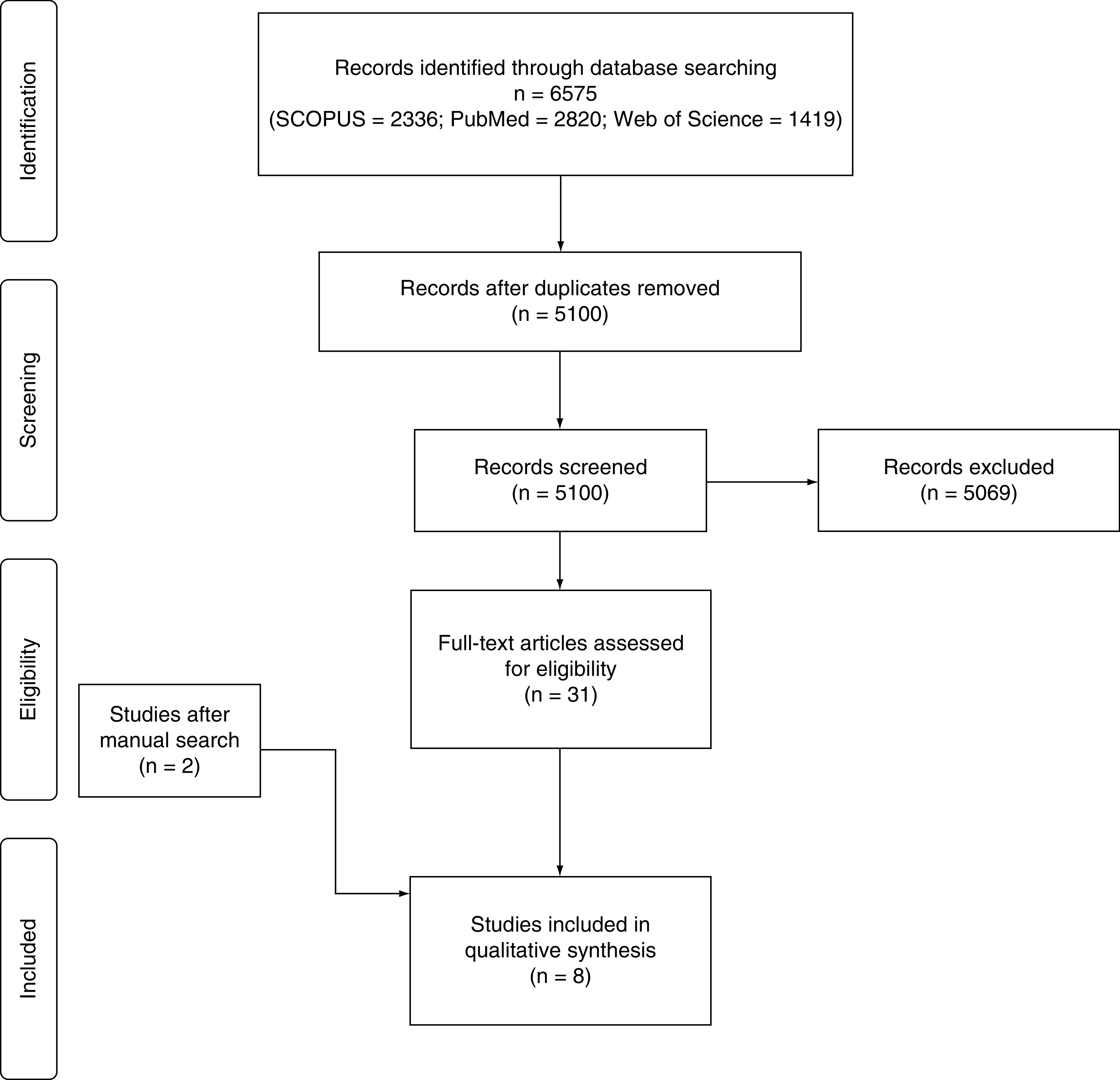
PRISMA flow diagram of the study selection process. PRISMA: Preferred Reporting Items for Systematic Reviews and Meta-Analyses.

Our literature search yielded 5100 unique records. Following the title and abstract screening, 31 studies remained eligible for full-text screening. Finally, eight cohort studies (n = 19,399 patients) were eligible for inclusion in this meta-analysis [19–26]. The flow diagram of the study selection process is shown in Figure 1.

### Baseline characteristics of included studies

We included eight studies with a total population of 19,399 COVID-19 patients, including 288 with AIS versus 19,111 without AIS. Three studies were conducted in China [20–22], one in Spain [19], one in Italy [23], two in the USA [24,25] and one was multinational [26]. The summary of the included studies and their population characteristics are shown in Table 1.

**Table 1.** Summary of the included studies and their population characteristics.

Study	Type of study	Country	Patients (n)	Age (years), mean (± SD)		Gender (males)		Medical history		Severe COVID-19	
				AIS	Non-AIS	AIS	Non-AIS	AIS	Non-AIS	AIS	Non-AIS
Annie *et al.* (2020)	Retrospective observational study	USA	AIS = 64 non-AIS = 9294 total = 9358	39.3 (± 9.0)	36.7 (8.5)	25	3680	Smoking (22), HTN (39), DM (21), obesity (30)	Smoking (548), HTN (1087), DM (604), obesity (1617)	NA	NA
Fan *et al.* (2020)	Retrospective observational study	China	AIS = 6, non-AIS = 80 total = 86	68.2 (± 2.1)	66.5 ± 11.5	5	49	Smoking (1), HTN (3), DM (2)	Smoking (11), HTN (41), DM (17)	NA	NA
Hernández-Fernández *et al.* (2020)	Retrospective observational study	Spain	AIS = 17, non-AIS = 1667 total = 1684	68.2 (± 13.0)	NA	13	NA	Smoking (2), HTN (10), DM (6), dyslipidemia (7)	NA	NA	NA
Zhang *et al.* (2020)	Single-center case series	China	AIS = 39, non-AIS = 612 total = 651	Median (IQR) 70 (67–84)	Median (IQR) 55 (37–67)	33	259	HTN (43), DM (19)	HTN (183), DM (93)	6	167
Li *et al.* (2020)	Retrospective observational study	China	AIS = 10, non-AIS = 209 total = 219	75.7 (± 10.8)	52.1 (15.3)	5	83	Smoking (3), HTN (9), DM (6), heart disease (3), malignancy (1)	HTN (46), DM (25), heart disease (14), malignancy (13)	9	83
Lodigiani *et al.* (2020)	Retrospective observational study	Italy	AIS = 9, non-AIS = 353 yotal = 362	NA	NA	NA	NA	NA	NA	3	58
Bach *et al.* (2020)	Retrospective observational study	USA	AIS = 20, non-AIS = 663 total = 683	63 (± 10.7)		13	384	Smoking (5), HTN (18), DM (13), dyslipidemia (8), obesity (17)	NA	NA	NA
Shahjouei *et al.* (2020)	Retrospective observational study	Multiple countries	AIS = 123, non-AIS = 6233 total = 6356	68.6 (13.9)	58 (14)	67	3688	Smoking (15), HTN (61), DM (32)	Smoking (385), HTN (1912), DM (1312)	NA	NA

AIS: Acute ischemic stroke; DM: Diabetes milletus; HTN: Hypertension; IQR: Interquartile range; NA: Not available.

### Quality of the included studies

As assessed by the NOS, the included studies' overall quality was acceptable (Table 2). Regarding selection bias, all studies had good quality in the exposed cohort's representativeness, selection of the nonexposed cohort and ascertainment of exposure. Four studies did not clarify whether the outcome was presented initially or not [22,24–26]. As for comparability, all studies presented comparable cohorts except Annie *et al.* [24]. All studies showed good quality in outcome assessment, sufficiently long follow-up and the cohort's adequacy.

**Table 2.** Newcastle Ottawa Scale quality assessment of the included studies.

Study	Selection				Comparability	Outcomes		
	Representativeness of the exposed cohort	Selection of the nonexposed cohort	Ascertainment of exposure	The outcome was not present at the start of the study	Comparable cohort	Assessment of outcome	Follow-up long enough	Adequacy of follow-up of the cohort
Hernández-Fernández *et al*.(2020)	*	*	*	*	*	*	*	*
Li *et al.* (2020)	*	*	*	*	*	*	*	*
Lodigiani *et al.* (2020)	*	*	*	*	*	*	*	*
Zhang *et al.* (2020)	*	*	*	*	*	*	*	*
Annie *et al.* (2020)	*	*	*	–	–	*	*	*
Fan *et al.* (2020)	*	*	*	–	*	*	*	*
Shahjouei *et al.* (2020)	*	*	*	–	*	*	*	*
Bach *et al.* (2020)	*	*	*	–	*	*	*	*

### Mortality rates

**Figure 2.**
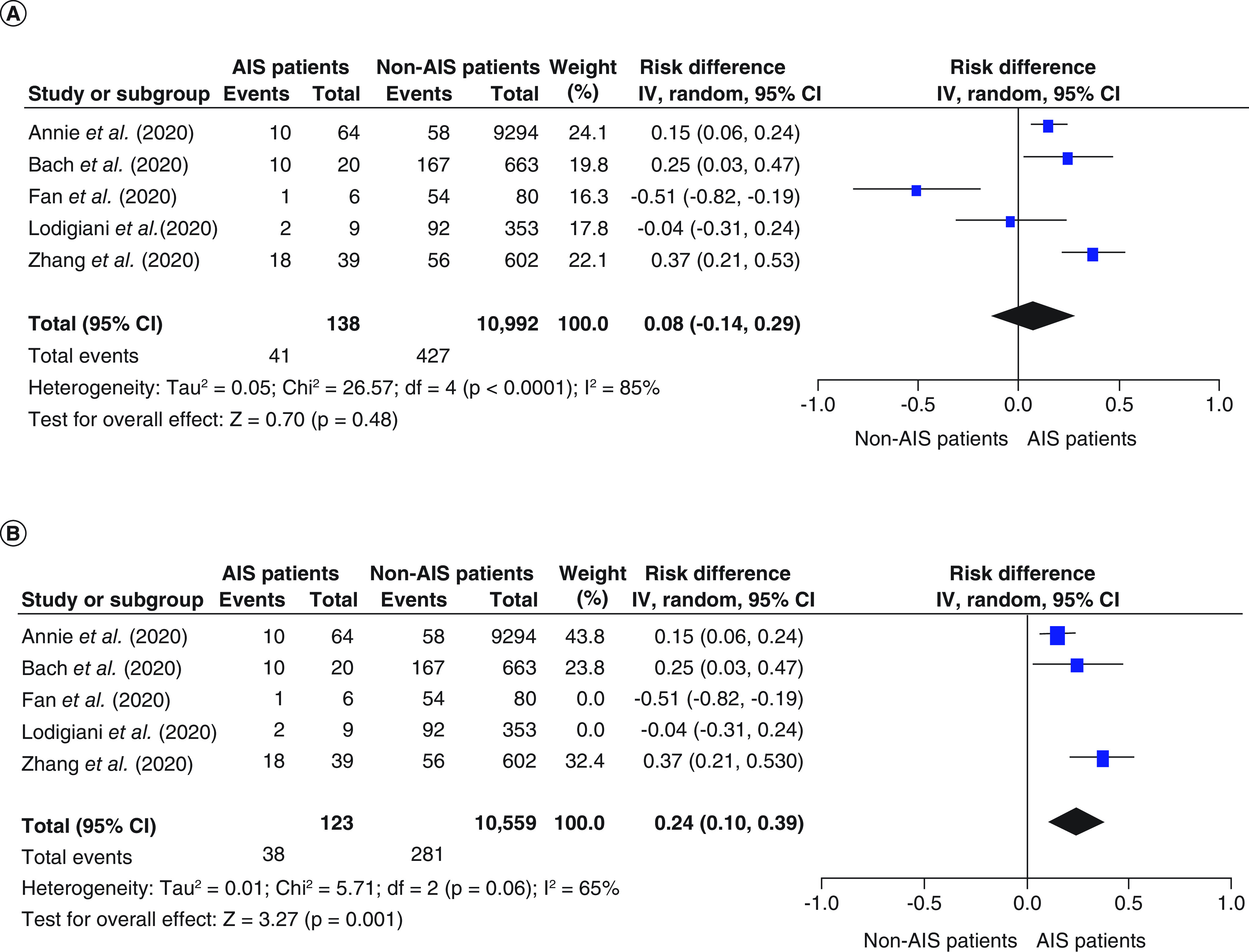
The forest plot of the risk difference in mortality and the corresponding 95% CIs (right graph area suggests higher mortality in the COVID-19 with AIS group). AIS: Acute ischemic stroke; df: Degrees of freedom.

Six studies reported the mortality rate in both COVID-19 with AIS and COVID-19 without AIS patients [20–25]. Mortality rates tended to be higher in the COVID-19 with AIS group than the COVID-19 without AIS group; however, the pooled effect estimate was not statistically significant (RD: 0.08; 95% CI: -0.14–0.29; p = 0.48; Figure 2A). Pooled studies were not homogenous (p < 0.001, I^2^ = 85%). After doing sensitivity analysis by excluding small studies (those reporting data from fewer than 10 patients with COVID-19 with AIS), heterogeneity was resolved, and the overall risk difference showed higher mortality in the COVID-19 with AIS group compared with the COVID-19 without AIS group (RD: 0.24; 95% CI: 0.10–0.39; p = 0.001; Figure 2B). The COVID-19 with AIS group's overall mortality rate was 29.6% compared with 2.6% in the COVID-19 patients without AIS.

### Laboratory findings

**Figure 3.**
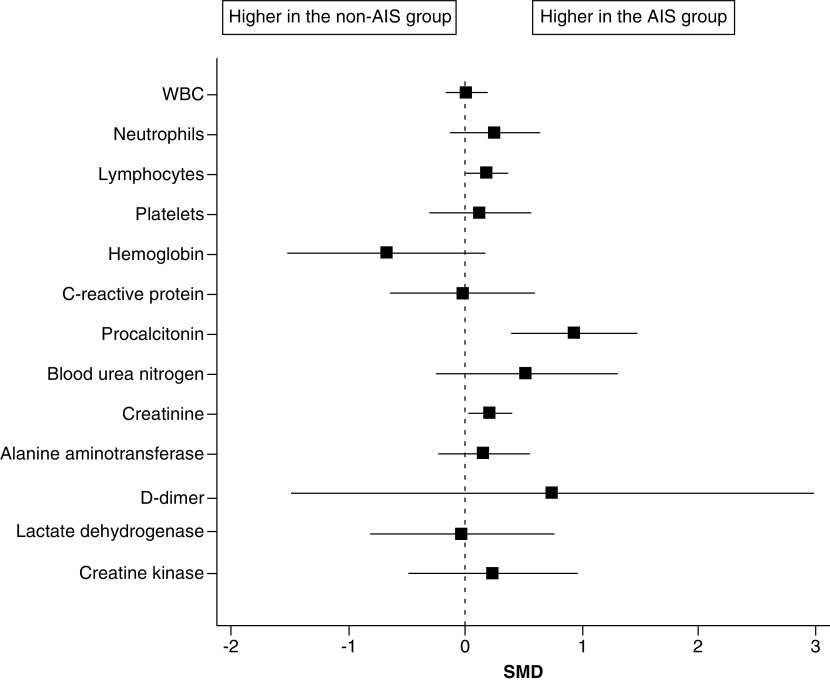
The forest plot of the SMD and the corresponding 95% CI for the laboratory findings (SMD >0 suggests higher values in the COVID-19 with A IS group). AIS: Acute ischemic stroke; SMD: Standardized mean difference.

Two studies compared biomarker levels between COVID-19 with AIS (n = 129) and COVID-19 without AIS patients (n = 6,280) [22,26]. When data of the laboratory findings were pooled in the meta-analysis model, the overall SMD between the two groups did not favor either group in terms of the white blood cells, hemoglobin, platelets, C-reactive protein, blood urea nitrogen, alanine aminotransferase, d-dimer, lactate dehydrogenase or creatine kinase. Patients in the COVID-19 with AIS group had significantly higher lymphocyte count (SMD: 0.19; 95% CI: 0.02–0.36, p = 0.03), procalcitonin level (SMD: 0.94; 95% CI: 0.41–1.47; p < 0.001), and creatinine level (SMD: 0.21; 95% CI: 0.04–0.39; p = 0.02) compared with those in the COVID-19 without AIS group (Figure 3 & Table 3).

**Table 3.** The effect size and statistical significance of the laboratory findings.

	Effect size	CI	p-value
White blood cells	0.01	(-0.16–0.18)	0.91
Neutrophils	0.25	(-0.12–0.63)	0.19
Lymphocytes	0.19	(0.02–0.36)	0.03
Platelets	0.13	(-0.3–0.56)	0.54
Hemoglobin	-0.67	(-1.5–0.16)	0.11
C-reactive protein	-0.02	(-0.62– 0.58)	0.94
Procalcitonin	0.94	(0.41–1.47)	<0.001
Blood urea nitrogen	0.52	(-0.25–1.29)	0.18
Creatinine	0.21	(0.04–0.39)	0.02
Alanine aminotransferase	0.16	(-0.22–0.55)	0.40
D-dimer	0.75	(-1.48–2.97)	0.51
Lactate dehydrogenase	-0.03	(-0.81–0.76)	0.95
Creatine kinase	0.24	(-0.47–0.95)	0.51

## Discussion

Overall, 19,399 COVID-19 patients were included in the qualitative and quantitative evidence synthesis. This study shows that COVID-19 patients who developed AIS were at a 24% higher risk of mortality than non-AIS COVID-19 patients. In addition, specific laboratory values as lymphocyte count, procalcitonin level and creatinine level were higher in the COVID-19 with AIS group. There was no significant difference in the remaining laboratory values, which could be explained by the limited number of available studies.

The high mortality rate in COVID-19 with AIS group in our meta-analysis (29.6%) is concordant with recent studies on COVID-19 [27–29]. A recent systematic review showed that the proportion of patients with COVID-19 who developed stroke was 1.8% (95% CI: 0.9–3.7%), and the in-hospital mortality rate was 34.4% (95% CI: 27.2–42.4%) [27]. However, this study is different from our meta-analysis in that it included patients with all types of stroke and not only ischemic stroke. It also did not directly compare the mortality of COVID-19 patients with stroke versus those without stroke [27]. In a recent systematic review by Tan *et al*. investigating the clinical features, neuroimaging findings and outcomes of AIS in COVID-19 patients, the pooled incidence of AIS in COVID-19 patients from observational studies was 1.2%, with a high mortality rate of 38% [28]. This study did not include a direct comparison between COVID-19 with AIS against those without AIS. Dmytriw *et al.* investigated the effects of race differences among COVID-19 with AIS patients and found that the mortality rate was significantly higher in African–Americans than other races (55.6% vs 28.6%; p = 0.05) [29]. Another study by Yaghi *et al*. found that 0.9% of all COVID-19 hospitalized patients experienced an ischemic stroke, and these patients had a higher inpatient mortality rate than contemporary and historical ischemic stroke controls before the COVID-19 pandemic (63.6% vs 9.3%; p < 0.001) [30]. Even patients with a history of stroke before their COVID-19 infection had higher mortality, as evidenced by a recent study in Wuhan, China, on 1875 patients, which found that patients with a history of stroke had worse outcomes compared with those without a history of stroke, with lower rates of discharge and higher mortality risks (24.0 vs 43.3%, 14.0 vs 8.3%, respectively) [31].

Compared with the previously reported mortality rates of ischemic stroke patients in the general population, COVID-19 patients with AIS in our meta-analysis had a higher mortality rate (29.6%) [15,32,33]. Saposnik *et al*. reported the mortality rates for 12,262 AIS patients ranged from 12.2 to 12.6% at 30 days and 22.5 to 22.9% at 1 year [15]. A previous systematic review and meta-analysis showed that from 2000 to 2008, early (21 days to 1 month) case fatality of ischemic stroke ranged from 13 to 23% in high-income countries and from 13 to 19% in low to middle-income countries [32]. Ganesh *et al*. conducted a retrospective study on 319,972 stroke/TIA hospitalized patients and found that the crude 30-day mortality rate decreased from 15.8% in 2003/2004 to 12.7% in 2012/2013 in provinces with stroke systems while remained 14.5% in those without such systems [33]. By 8 April 2020, the total number of confirmed COVID-19 cases globally was 133.9 million, with 2.9 million deaths (mortality rate of 2.2%) [34]. A systematic review and meta-analysis of published evidence on COVID-19 until July 2020 showed that the COVID-19 infection fatality rate across populations was 0.68% (0.53–0.82%) [35]. Thus, this meta-analysis shows that the mortality rate of COVID-19 patients with AIS is greater than the mortality rate of either COVID-19 patients in general or patients with acute ischemic stroke alone, suggesting a compounded mortality effect of stroke and COVID-19 when they co-occur.

Regarding laboratory findings, the difference between COVID-19 with AIS and COVID-19 without AIS was only significant for lymphocyte count, procalcitonin and creatinine, which were significantly higher in AIS patients. Creatinine level is one of the most critical indicators that correlates with ischemic and hemorrhagic strokes [36]. Snarska *et al*. have investigated the impact of kidney functions on in-hospital patients with ischemic and hemorrhagic strokes. They have found that in both, creatinine was a predictor for stroke outcome [36]. Our analysis of the available literature has shown that two studies [22,26] have assessed creatinine levels for COVID-19 stroke patients. The meta-analysis pooled results show that creatinine levels among COVID-19 with AIS patients were higher than the COVID-19 without AIS group. This finding suggests that patients with COVID-19 and high creatinine levels should be closely monitored for the development of any focal neurologic deficit that might indicate the occurrence of AIS for timely intervention. Current literature shows that severe COVID-19 is associated with lymphopenia [37,38]. The two studies included in the meta-analysis that compared lymphocyte count between COVID-19 patients with AIS against those without AIS showed low or low normal lymphocyte count in both groups, but absolute lymphocyte count was higher in COVID-19 with AIS group [22,26]. Elevated procalcitonin has been reported in patients with severe COVID-19, stroke with malignant cerebral edema, and stroke-associated pneumonia [39–41]. Scattered reports have documented an increase in procalcitonin in COVID-19 patients with AIS, with which findings of our study corroborate [42–45]. Other biomarkers, such as elevated D-dimer and fibrinogen, as well as detection of antiphospholipid antibodies, were reported to be significantly associated with AIS developing in COVID-19 patients [46].

**Figure 4.**
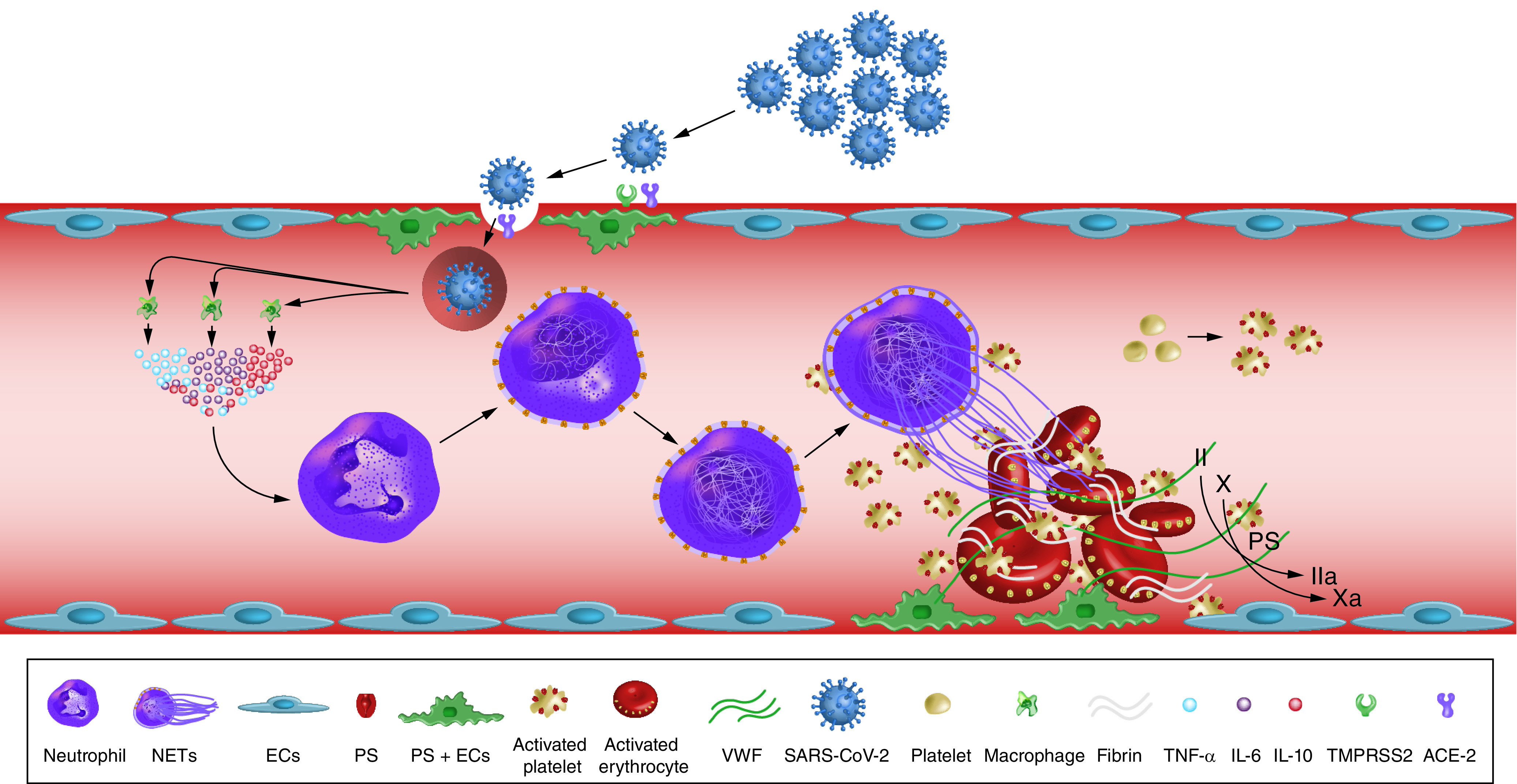
Mechanisms of COVID-19-associated hypercoagulability. SARS-CoV-2 enters blood vessels through the ACE2 receptors on endothelial cells. Viral infection activates monocytes and macrophages to release a proinflammatory cytokine storm. This results in neutrophil activation and recruitment and tissue factor activation. Persistent neutrophil recruitment promotes a NET. NET and SARS-CoV-2 infection induce an endothelial cell injury, releasing vWF and activate platelets. The NET formation, TF activation and platelet aggregation induce coagulopathy and inhibit fibrinolysis. These processes represent the most recent data describing COVID-19-associated hypercoagulability. ACE2: Angiotensin-converting enzyme 2; EC: Endothelial cell; NET: Neutrophil extracellular trap; SARS-CoV-2: Severe acute respiratory syndrome coronavirus 2; TF: Tissue factor; vWF: von Willebrand factor. Redrawn from [49].

**Figure 5.**
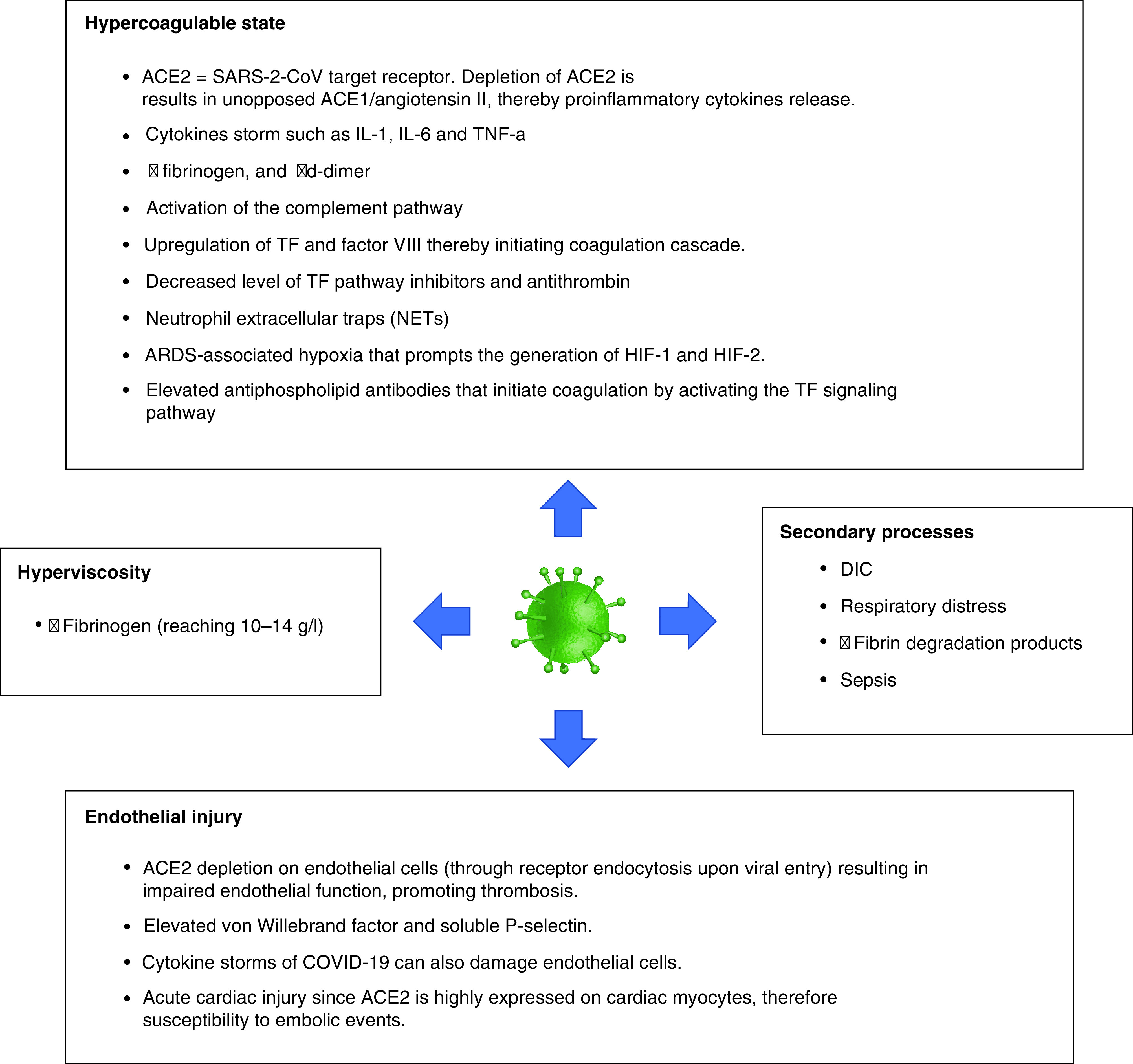
Proposed mechanisms thought to result in acute ischemic stroke in COVID-19 patients. ACE: Angiotensin-converting enzyme; DIC: Disseminated intravascular coagulation; HIF: Hypoxia-inducible factor; TF: Tissue factor.

Several studies indicated an association between COVID-19 and increased arterial and venous thromboses rates [47–63]. Reported rates of arterial thrombosis range from 2.8 to 3.8% [47]. Multiple mechanisms could explain the increased risk of ischemic strokes in COVID-19 patients. These include depletion and viral uptake of angiotensin-converting enzyme 2 (ACE2) receptors [48], hypercoagulability (Figure 4) [49–51], high systemic inflammatory response or ‘cytokine storm’ [52], neutrophil extracellular traps (NETs) [53,54], complement system activation [55], increased expression of tissue factor on endothelial cells and infiltrating macrophages and neutrophils [56], hypoxia [57], elevated antiphospholipid antibodies [58], elevated vWF activity, tissue factor, and factor VIII levels [59,60], vascular endothelial injury [61], hyperviscosity [62] and cardiac injury resulting in cerebral embolism [63]. Figure 5 summarizes the proposed mechanisms of AIS in COVID-19 patients.

Our meta-analysis is limited by the relatively small number of available studies, the small sample size in some reports and a limited number of available parameters compared between groups. This is because studies did not consistently report AIS outcomes in the literature, and only a few studies provided head-to-head comparisons between COVID-19 with AIS and COVID-19 without AIS populations. Pooled studies were not homogenous, which was resolved by doing sensitivity analysis, excluding small studies. Although there might be a suggestion that mortality in COVID-19 with AIS was not necessarily higher due to AIS per se but rather the severity or co-occurring complications of COVID-19 such as renal failure and pneumonia, the included studies' data did not consistently provide such manifestations, complications or severity of COVID-19 for their patients, so including such variables in the meta-analysis was not possible. However, because this study compares groups of patients (COVID-19 with AIS vs those without AIS), the aim was not to establish direct causation between AIS and mortality. Another limitation is that only two studies investigated laboratory findings associated with AIS in COVID-19 patients [22,26]. Although Fan *et al*.'s study had a substantially small sample size of six AIS patients compared to 80 COVID-19 patients without AIS [22], this was compensated by Shahjouei *et al.* large sample size with a relatively higher number of COVID-19 with AIS patients (n = 123) [26].

Nonetheless, this meta-analysis expands the literature by providing evidence on the increased mortality in COVID-19 patients with AIS, which requires medical attention and prompt management to reduce the mortality.

## Conclusion

COVID-19 patients with AIS have higher rates of mortality compared with COVID-19 patients without AIS. There is a suggestion that COVID-19 with AIS patients have higher levels of lymphocytes, procalcitonin and creatinine than those without AIS. Future prospective, well-designed studies should provide more data on the predictors of mortality among COVID with AIS patients. Also, future clinical trials are needed to evaluate interventions to reduce mortality in this population.

## Future perspective

On the basis of this study's findings, the management of COVID-19 patients should follow a risk stratification model. The healthcare for COVID-19 patients who developed AIS or at risk of developing AIS should be maximized to lower their mortality. Specific biomarkers such as lymphocyte count and levels of procalcitonin and creatinine should be monitored closely as they can predict the occurrence of AIS in COVID-19 patients. Future prospective, well-designed studies should provide more data on the predictors of mortality among this population. Future clinical trials are needed to evaluate interventions to reduce mortality in COVID-19 with AIS patients.

**Summary points**

- COVID-19 is an evolving disease with varying clinical courses, ranging from asymptomatic infection to severe complications, including death.
- Previous reports indicated an association between COVID-19 and inflammatory coagulopathy that causes disseminated vascular obstructions, including but not limited to acute ischemic stroke (AIS).
- This systematic review and meta-analysis aimed to compare the mortality rates of COVID-19 patients who developed AIS versus those who did not and determine the significant demographic, clinical and laboratory characteristics of COVID-19 patients who developed AIS.
- A total of eight studies with 19,399 COVID-19 patients, including 288 with AIS versus 19,111 without AIS, were included in this study.
- Six studies reported the mortality rates in both COVID-19 with AIS and COVID-19 without AIS patients. Two studies compared biomarkers' levels between COVID-19 with AIS (n = 129) and COVID-19 without AIS patients (n = 6,280).
- The meta-analysis showed higher mortality in the COVID-19 with AIS group compared with the COVID-19 without AIS group (RD: 0.24; 95% CI: 0.10–0.39; p = 0.001).
- Higher lymphocytes, procalcitonin and creatinine levels were seen in COVID-19 patients with AIS compared with those without AIS.
- The overall standardized mean difference between the two groups did not favor either of the two groups in terms of the white blood cells, hemoglobin, platelets, C-reactive protein, blood urea nitrogen, alanine aminotransferase, D-dimer, lactate dehydrogenase or creatine kinase.
- COVID-19 patients with AIS in our meta-analysis had a higher mortality rate than previously reported mortality rates of AIS patients in the general population.
- In conclusion, COVID-19 patients who develop AIS have higher mortality than COVID-19 patients without AIS; thus, they need timely interventions to lower their mortality. The biomarkers mentioned herein should be monitored closely in COVID-19 patients as they are associated with AIS development.
